# P-1025. Outcome Analysis of Breakthrough Invasive Aspergillosis on Anti-Mold Azole Prophylaxis: Thirty Year Experience in Hematologic Malignancy Patients

**DOI:** 10.1093/ofid/ofae631.1215

**Published:** 2025-01-29

**Authors:** Hiba Dagher, Anne-Marie Chaftari, Andrea Haddad, Ying Jiang, Jishna Shrestha, Robin Sherchan, Peter Lamie, Jennifer Makhoul, Patrick Chaftari, Ray Y Hachem, Issam I Raad

**Affiliations:** UT MD Anderson Cancer Center, Houston, Texas; MD Anderson UT, Houston, Texas; MD Anderson Cancer Center, Houston, Texas; The University of Texas MD Anderson Cancer Center, Houston, Texas; MD Anderson Cancer Center/ Baylor College of Medicine, Houston, Texas; MD Anderson Cancer Center / Baylor College of Medicine, Houston, Texas; UT MD Anderson Cancer Center, Houston, Texas; University of Texas Health Science Center at Houston/MD Anderson Cancer Center, Houston, TX; UT MDAnderson Cancer Center, Houston, TX; MD Anderson UT, Houston, Texas; MD Anderson UT, Houston, Texas

## Abstract

**Background:**

In patients with haematologic malignancy (HM) and stem cell transplant recipients, Invasive Aspergillosis (IA) remains associated with a poor prognosis. Anti-mold azoles have improved the outcome of IA when used therapeutically but are extensively used as prophylaxis. There is limited data regarding the outcomes of HM patients with IA breakthroughs on anti-mold azoles. We aimed to determine whether breakthrough IA patients on anti-mold azole prophylaxis are associated with a worse outcome.

**Methods:**

We compared baseline characteristics, antifungal therapies, and outcomes in patients with breakthrough IA between July 1993 and July 2023 who received anti-mold azole prophylaxis (group 1) versus anti-mold non-azole prophylaxis (echinocandins) (group 2) versus no anti-mold prophylaxis (group 3). We compared mortality and clinical response at 6 and 12 weeks after diagnosis.

**Results:**

614 patients with IA were analyzed, 163 in group 1, 62 in group 2 and 389 in group 3. 42-Day all-cause mortality (45%) and IA-associated mortality (35%) were significantly higher in group 1 as compared to group 3 (33% p=0.032 & 24% p=0.02 respectively). Breakthrough IA patients in group 1 were treated with Ampho B based (AMB) primary therapy at a significantly higher rate (64%) as compared to group 3 (44% p< 0.001). By multivariate analysis, AMB primary therapy was independently associated with worse response at EOT (OR= 0.64 p=0.041) and all-cause 84-day mortality (OR=1.85 p=0.002). Interestingly, anti-mold azole prophylaxis was independently associated with IA-associated and all-cause 42-day mortality (OR=1.85 p=0.009 and OR=1.70 p=0.013, respectively). Anti-mold azole primary therapy was independently associated with a successful response at EOT, protective against IA-associated and all-cause 42-day mortality, and IA- associated and all-cause 84-day mortality (p= < 0.01).

**Conclusion:**

Breakthrough IA patients receiving anti-mold azole prophylaxis are associated with a worse prognosis possibly because they are more likely switched to AMB primary therapy which is associated with a poor outcome compared to anti-mold azole therapy.

**Disclosures:**

**All Authors**: No reported disclosures

